# Supplementary material for: Fragment screening using biolayer interferometry reveals ligands targeting the SHP-motif binding site of the AAA+ ATPase p97
Source: Commun Chem. 2022 Dec 7;5:169. doi: 10.1038/s42004-022-00782-5 (PMC9814400; doi:10.1038/s42004-022-00782-5)
Supplement: Supplementary file 1 — Supplementary Information [file 42004_2022_782_MOESM1_ESM.pdf]

Supporting Information

“Fragment screening using biolayer interferometry reveals ligands targeting the SHP-motif binding site of the AAA+ ATPase p97“

Sebastian Bothe[1,2], Petra Hänzelmann[2], Stephan Böhler[1,2], Josef Kehrein[1], Markus Zehe[1], Christoph Wiedemann[3], Ute A. Hellmich[3,4,5], Ruth Brenk[6], Hermann Schindelin*[2] and Christoph Sotriffer*[1]

1 Institute of Pharmacy and Food Chemistry, University of Würzburg, Am Hubland, 97074 Würzburg, Germany

2 Rudolf Virchow Center for Integrative and Translational Bioimaging, University of Würzburg, Josef-Schneider-Str. 2, Haus D15, 97070 Würzburg, Germany

3 Faculty of Chemistry and Earth Sciences, Institute of Organic Chemistry and Macromolecular Chemistry, Biostructural Interactions, Friedrich Schiller University Jena, Humboldtstr. 10, 07743 Jena, Germany.

4 Cluster of Excellence “Balance of the Microverse”, Friedrich Schiller University Jena, Germany

5 Center for Biomolecular Magnetic Resonance (BMRZ), Goethe University Frankfurt, Max von Laue Str. 9, 60438 Frankfurt, Germany

6 Department of Biomedicine, University of Bergen, Jonas Lies Vei 91, 5020 Bergen, Norway

Supplementary Figures ……………….. 2

Supplementary Tables ……………….. 12

Supplementary Methods ……………….. 20

Chemical identity ……………….. 22

Supplementary References……………….. 23

Supplementary Figures

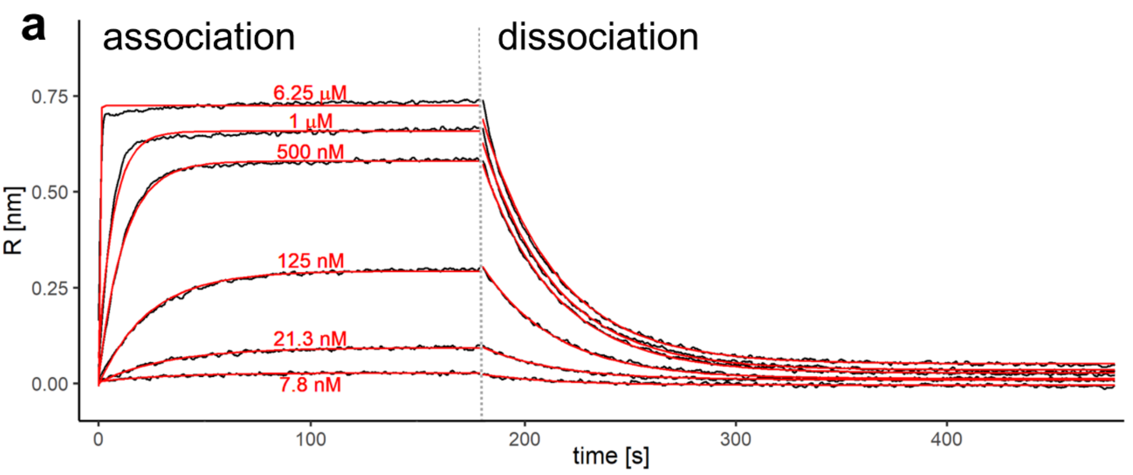

b

construct	p97-ND1 aa 1-480, ADP (BLI)	p97-ND1 aa 1-458, ADP (SPR) [1]	p97-ND1 aa 1-480, ADP (SPR) [1]
K_D^{steady} [nM]	200 ± 14	218 ± 3	98 ± 1
K_d^{kin} [nM]	239 ± 9	203 ± 2	93.8 ± 0.3
k_{on} x 10^5 [M^{-1} s^{-1}]	1.21 ± 0.02	2.42 ± 0.01	4.59 ± 0.02
k_{off} [s^{-1}]	0.0289 ± 0.0005	0.0491 ± 0.0003	0.0430 ± 0.0002

Supplementary Figure 1. Validation of BLI assay with p97-ND1. a Sensorgrams and the global fitting model of ADP as positive control for assay validation. The sensorgrams clearly follow a 1:1 global binding model. **b** For comparison, the reported affinity and kinetic constants for two similar ND1 constructs of p97 determined by Chou, T. F. *et al.* [1] using SPR are shown.

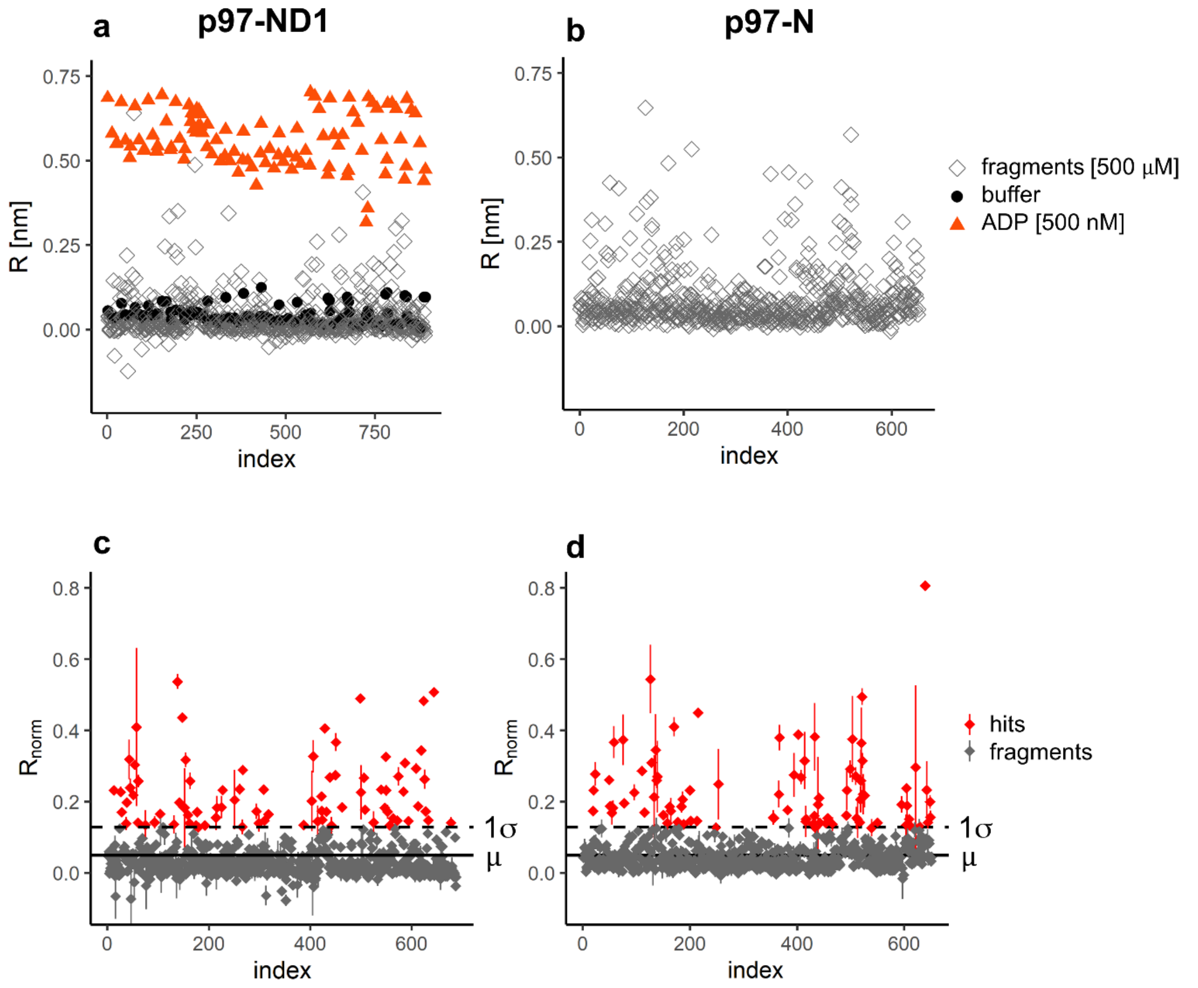

Supplementary Figure 2. Initial BLI screen with the ND1 domain (left panels) and the N-domain (right panels) of p97. a Results of the ND1 screen after double referencing. The signals of the ADP positive controls (red) and of the negative controls using assay buffer (black) are shown. Gray squares indicate the mean signal (n=2) of each measured fragment at a concentration of 500 µM. The numbers on the x-axis count fragments and positive controls and are therefore higher than in the subsequent diagrams with fragments only. **b** Results of the initial screen with the N-domain after double referencing and elimination of artifacts showing negative or extremely high values. Gray squares represent the mean signal (n=2) of each measured fragment at a concentration of 500 µM. **c** Normalized signals of the fragments of the ND1-domain screen with respect to the positive control, resulting in a mean normalized signal of 0.050 and a standard deviation of 0.079. **d** Normalized signals of the fragments of the N-domain screen with respect to the protein loading signals of the sensors being used, giving a mean value of 0.069 and a standard deviation of 0.080. A cut-off of one sigma was used in both cases (dashed lines). Error bars in **c** and **d** correspond to the range of the measured data points (n=2).

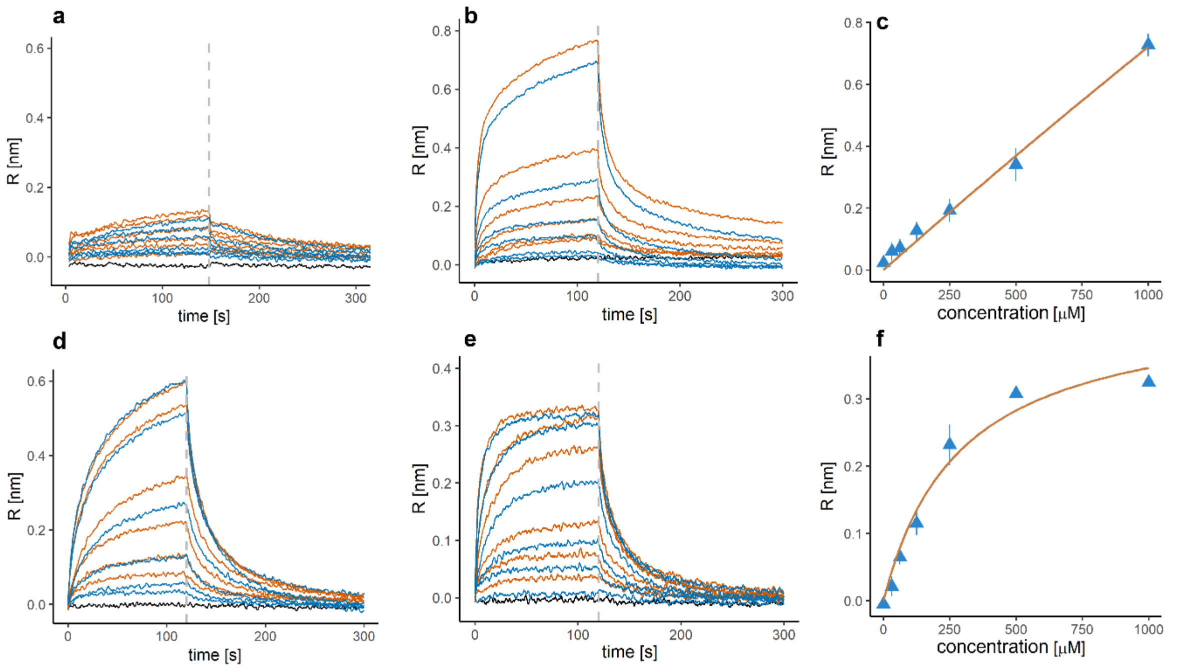

Supplementary Figure 3. Representative binding profiles for the interaction of four compounds with p97-N. Sensorgrams obtained with BLI from a 1:1 dilution series of 6 steps starting at a concentration of 1 mM. All concentrations were measured twice (first: orange, second: blue). The dashed line indicates the start of the dissociation phase. **a** Example of a compound exhibiting no significant signal. **b** Example of a compound showing a non-saturating response with increasing concentrations, which results **c** in a typical linear increase of the signal. In these cases, complete dissociation could not be reached. **d** Example of a fragment exhibiting a biphasic curve with no plateau phase at higher concentrations. **e** Sensorgram of one of the best binders. The curves reach a steady-state and show the typical shark fin profile. The analysis with a 1:1 Langmuir model **f** reveals the expected hyperbola characteristic of a single binding site exhibiting saturation at high concentrations.

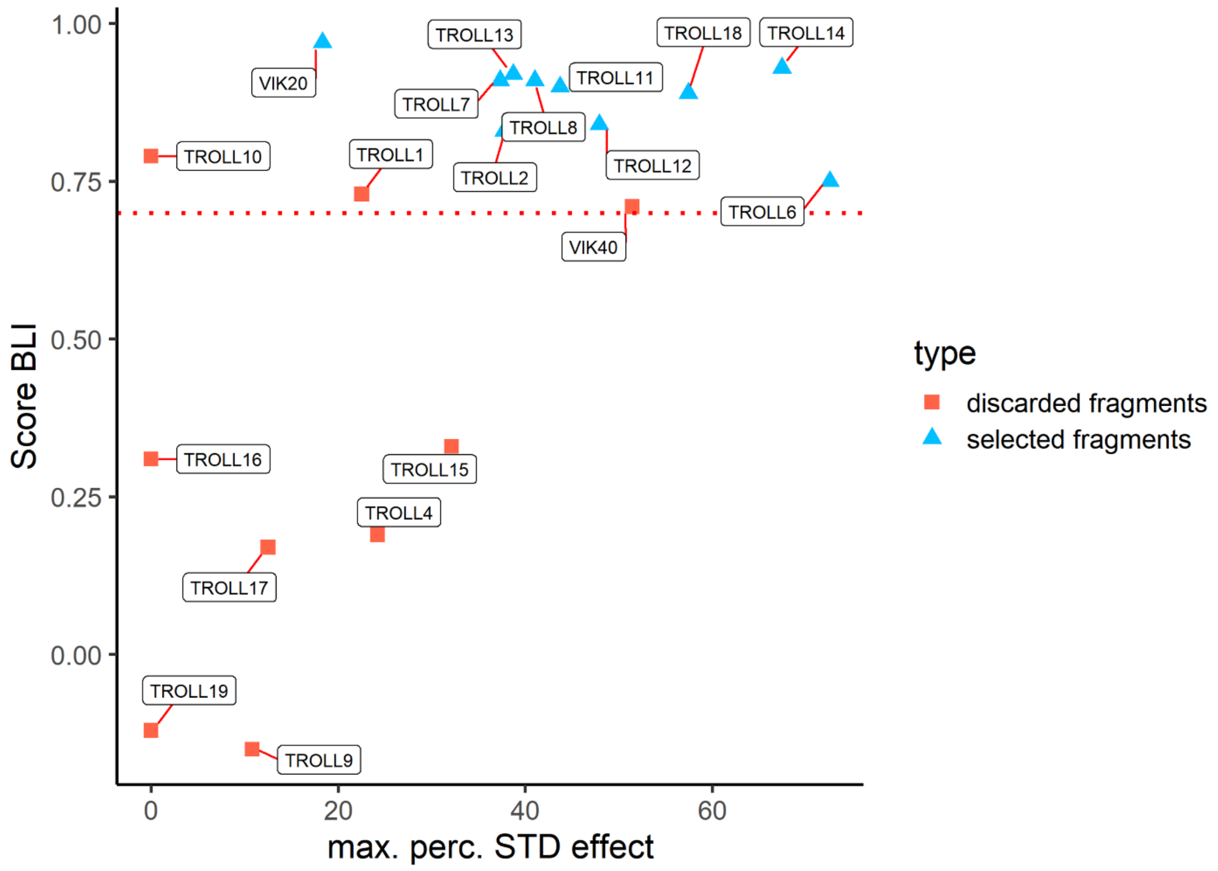

Supplementary Figure 4. Overview of calculated Score$_{BLI}$ and measured STD-effects of the fragments. Selected fragments are shown in blue and discarded molecules in red. Three fragments showed no STD-effect (TROLL10, TROLL16 and TROLL19). All other fragments displayed STD-effects between 10.5 and 72.5%. Selected fragments fulfill the criteria of a Score$_{BLI}$ value greater than 0.7. TROLL1 was discarded because no dose response with the p97 ND1-construct was obtained, VIK40 was discarded because of its low affinity (K_D > 1 mM) with the ND1-construct and TROLL10 because of its high discrepancy in affinity determined either by the kinetic analysis or in steady state measurements.

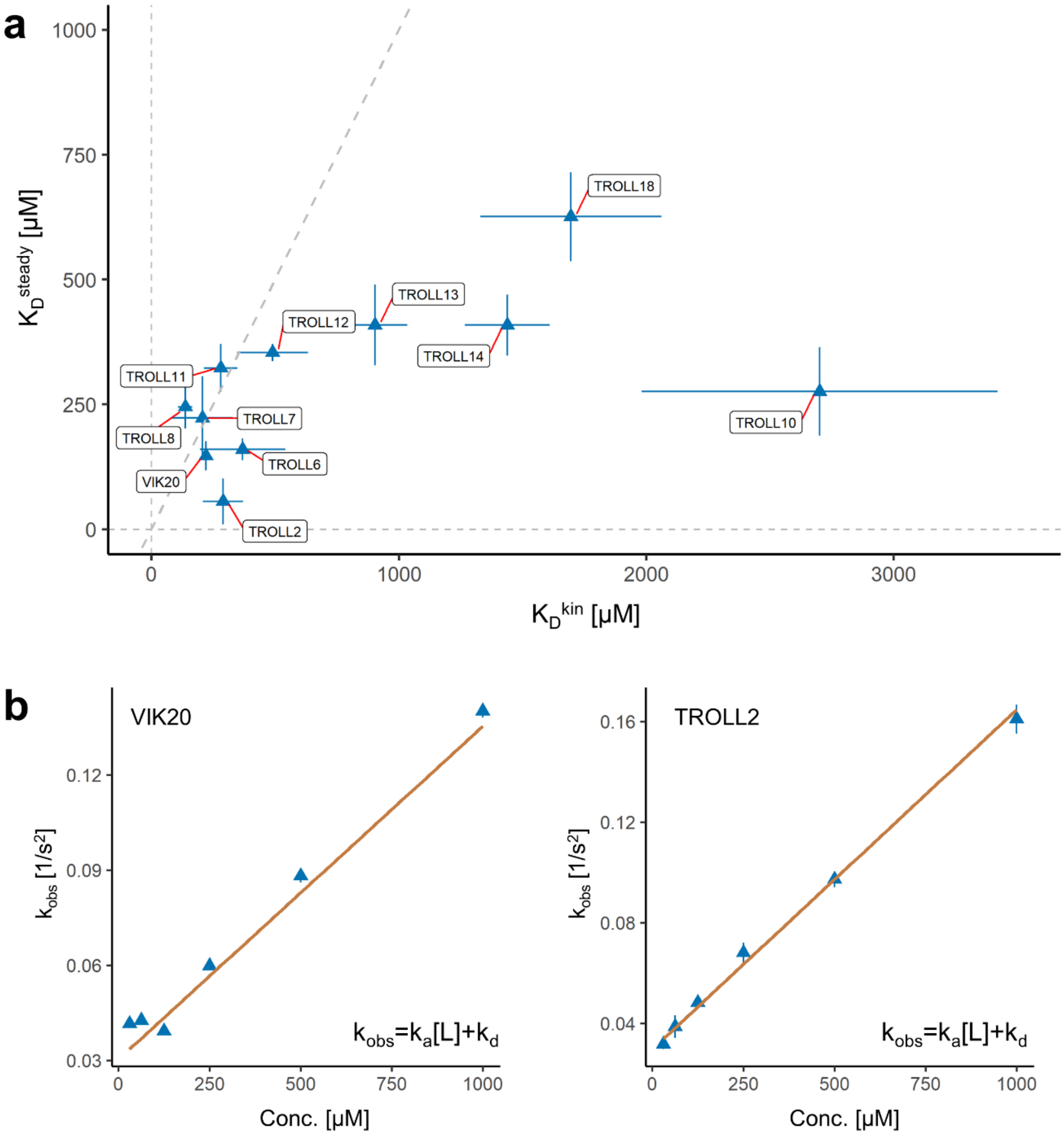

Supplementary Figure 5. Comparison of kinetic and steady state determined affinities of the fragments. Since the sensorgrams of the fragments showed slow on and off rates, the affinities were calculated based on the kinetic data of the dilution measurements. **a** Comparison of the kinetic affinity with the affinity determined by steady state. The diagonal shows the position of equal affinity values. Most fragments showed similar affinities. TROLL13, TROLL14 and TROLL18 showed an up to 3.5 times lower affinity based on the kinetic data. The highest discrepancy was found for TROLL10 resulting in a 10-fold lower affinity as derived from the kinetic data. Errors are derived from the fitting model. **b** The on and off rate constants were determined using the empirical rate constants and their dependency on the used concentration under the assumption of a 1:1 binding. Plotted are the results for TROLL2 and VIK20, which show a good agreement with the 1:1 binding model.

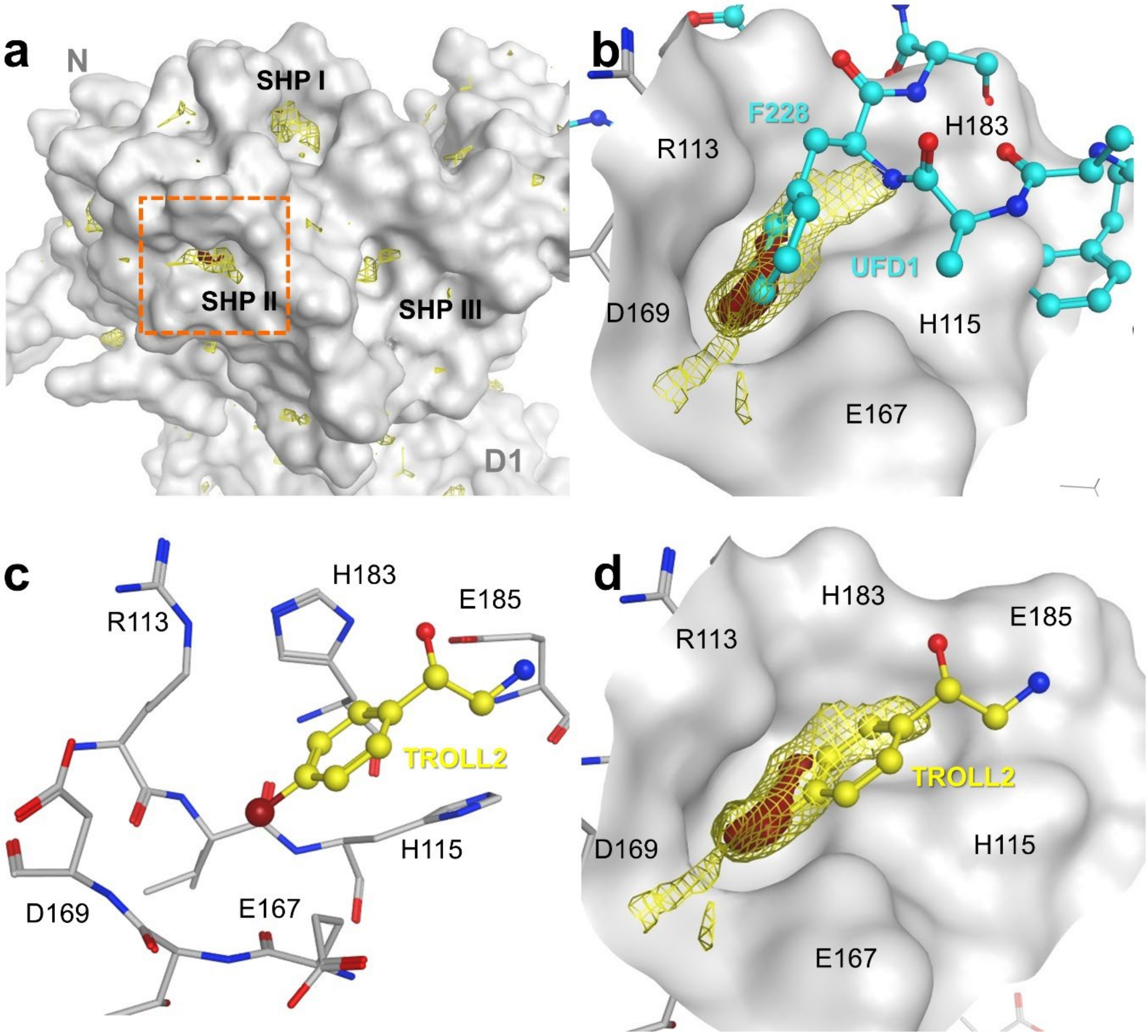

Supplementary Figure 6. Grid-based interaction analysis of the SHP II binding region. a The SHP binding site in the X-ray structure was analyzed using the grid-based interaction potential analysis function in MOE. The isosurfaces for a bromine atom and for an aromatic carbon atom probe are shown in dark red and yellow, respectively. The interaction analysis revealed aromatic hotspots in all three subpockets (SHP I, SHP II and SHP III). For the bromine atom only a hotspot within the SHP II is located. **b** Close up view of the SHP II binding pocket with the crystal structure of UFD1 (PDB: 5b6c) overlayed. The position of the consensus motif amino acid F228 of UFD1 (cyan) is in high agreement with the identified aromatic hotspot. **c** Close-up view of the binding region of TROLL2. TROLL2 interacts mainly with the hydrophobic parts of the amino acids R113 and E167 as well as with H183 and H115. Please note how the charged groups of R113, E167 and D169 are oriented away from the pocket, whereas the pocket itself is outlined by the alkyl chains of these residues. **d** Overlay of the interaction analysis with the X-ray binding pose of TROLL2. The identified bromine atom hotspot as well as the aromatic hot spot show a high agreement.

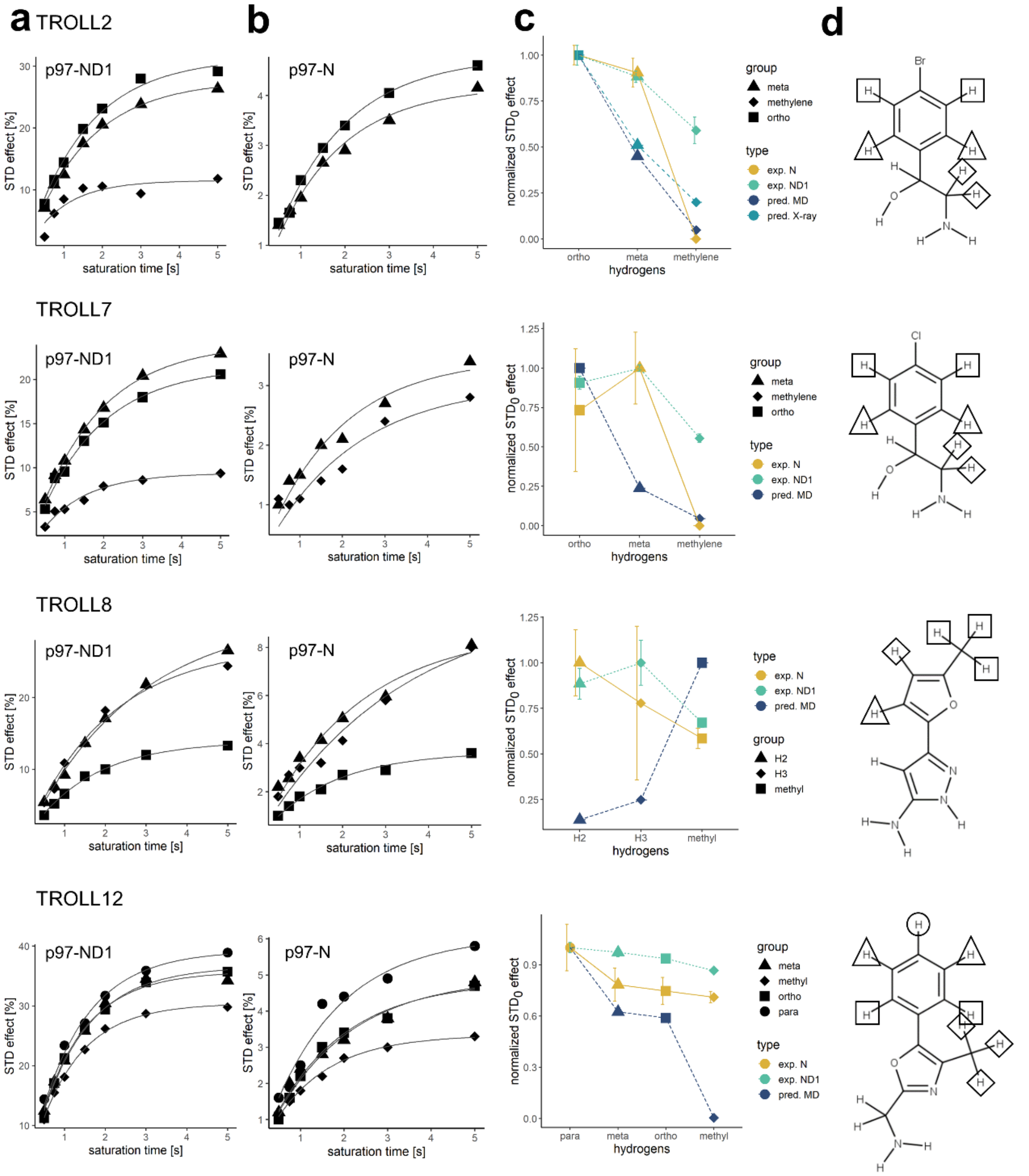

Supplementary Figure 7. Results of STD-NMR build-up measurements compared to predictions by CORCEMA-ST. Experimental STD-NMR build-up results for measurements with the ND1 construct **a** and the isolated N-domain **b** showing the mono-exponential fitting curves for STD_0 estimation of each fragment. **c** Comparison of the experimental effects with the predicted saturation effects using CORCEMA-ST based on the selected MD pose (as described in the Materials and Methods section) and, in case of TROLL2, based on the X-ray crystal structure. Due to the measurements with different constructs (hexameric ND1 and isolated N domain), the STD effects were normalized against the highest observed STD effect for better comparison. The error bars at the experimental data points indicate the error of the exponential fitting model. **d** Assignment of the signals for each fragment. Not all protons were experimentally accessible.

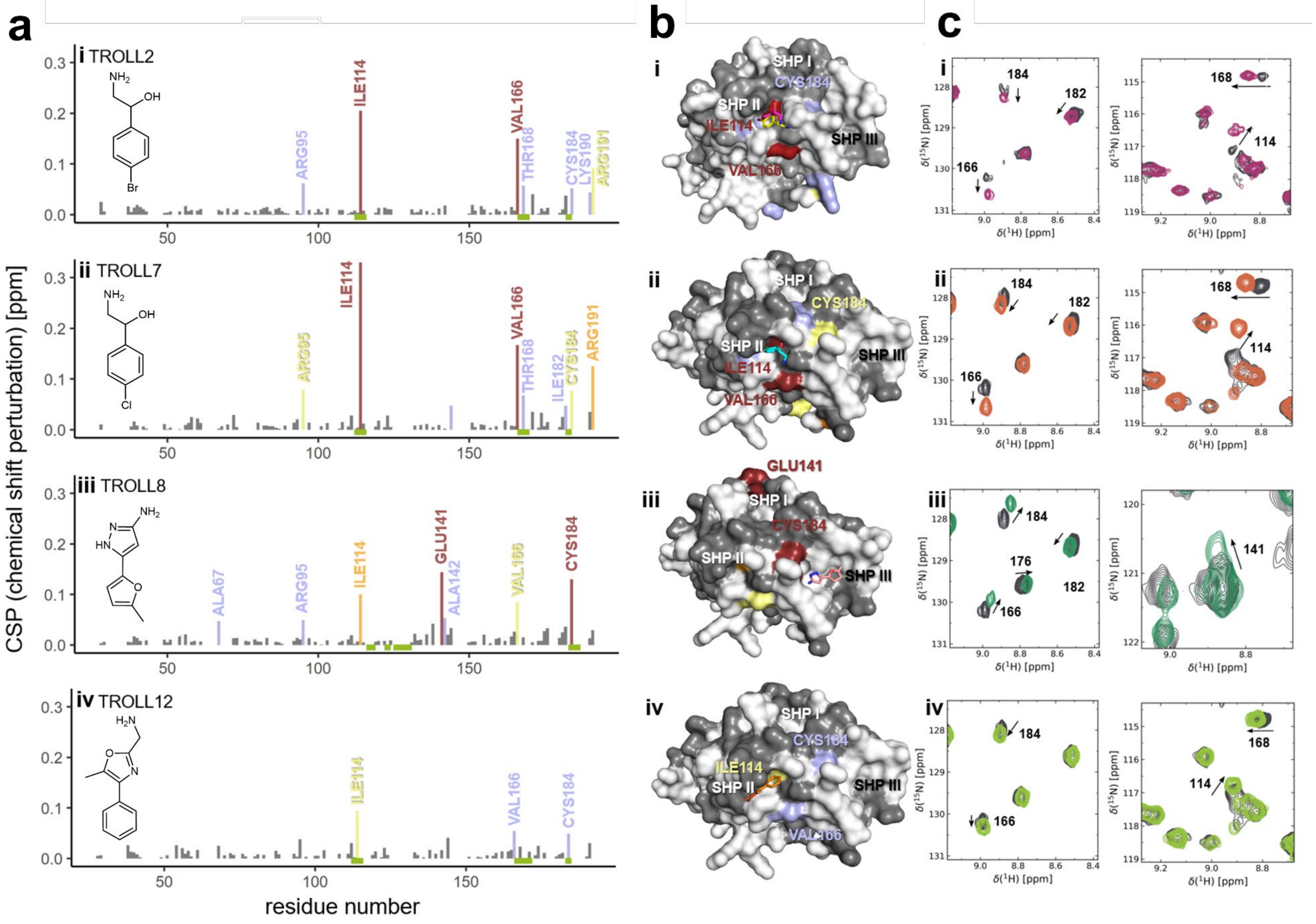
a
b
c
i TROLL2
ii TROLL7
iii TROLL8
iv TROLL12
CSP (chemical shift perturbation) [ppm]
residue number
ILE114
VAL166
ARG95
THR168
CYS184
LYS190
ARG191
ILE182
ALA67
GLU141
ALA142
SHP I
SHP II
SHP III
δ(¹H) [ppm]
δ(¹⁵N) [ppm]

Supplementary Figure 8. Chemical shift perturbation (CSP) observed upon titration of the p97 N-domain with the corresponding fragment. **a** Changes in the amide chemical shift are plotted for the residues in the p97 N-domain in terms of their strength corresponding to σ-levels of 4 (red), 3 (orange), 2 (yellow), 1 (blue). **b** Mapping of chemical shifts onto the surface of the N-domain together with the binding poses of the fragments (stick representation) received from the mixed-solvent MD simulations. **c** Selected regions of the ^{1}H-^{15}N-HSQC NMR spectra**.** Fragments in DMSO were titrated to ^{15}N-labeled p97-N (colored spectra)**.** The respective residue numbers are indicated based on the established backbone assignment [2]. The control spectra upon addition of an equal amount of DMSO are shown in gray.

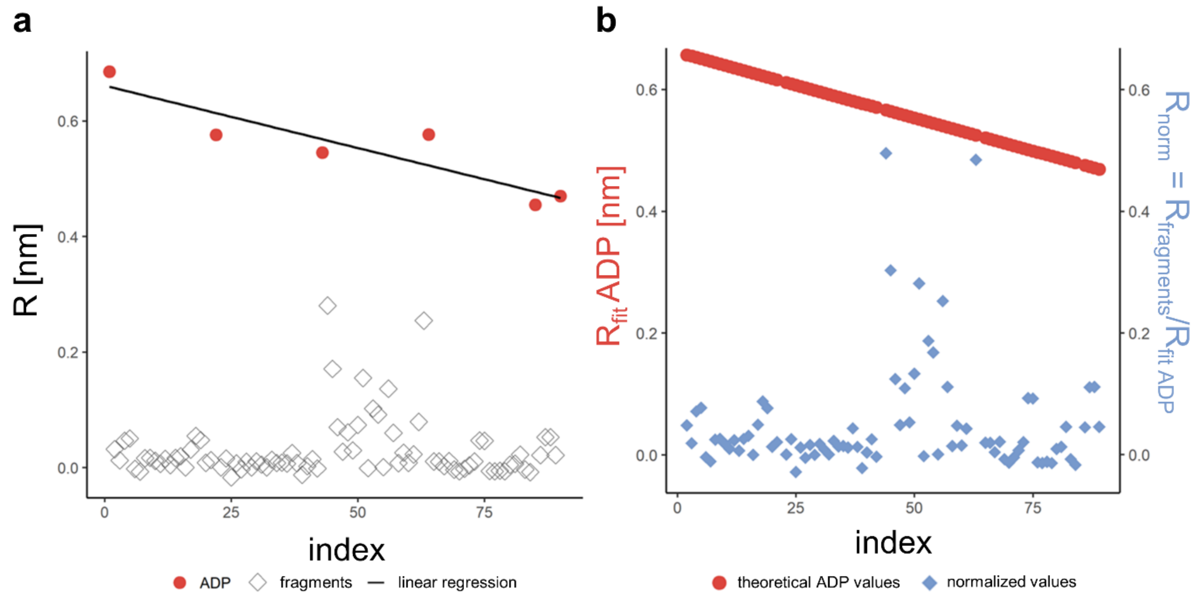

Supplementary Figure 9. Normalisation of signals for the ND1 screen. The signals of each sensor were corrected for partial inactivation of the protein over time and for different protein loading states of the sensors using 500 nM ADP as the positive control. Prior to normalization, the signals were aligned and double referenced using the Octet Analysis software. The normalization procedure is shown for one sensor: **a** With the signals of the ADP positive control (red dots) measured between each screening plate a linear regression was performed (black line). Grey diamonds reflect the signals of the fragments. **b** Using the resulting linear equation, the theoretically expected ADP signal at the time of measurement of each fragment was calculated (red dotted line). The normalization of the fragment signals was then carried out via $R_{norm}=R_{fragment}/R_{fit\ ADP}$ resulting in the normalized fragment values (blue diamonds).

Supplementary Tables

Supplementary Table 1. Data collection and refinement statistics for p97-ND1 in complex with TROLL2. (PDB entry: 7PUX)

**Data collection**	
Wavelength (Å)	0.9184
Resolution (Å)	47.87-1.73 (1.83-1.73) [a]
Space group	P622
Cell dimensions	
a=b, c (Å)	146.23, 84.40
α=β, γ (°)	90, 120
Unique reflections	42,850
<I/σ(I)>	16.68 (1.63) [a]
Multiplicity	26.47
Completeness *spherical* (%)	76.9 (25.31) [a]
Completeness *ellipsoidal* (%)	96.12 (82.0) [a]
CC(1/2)	0.999 (0.620) [a]
R_{pim}	0.027 (0.492) [a]
R_{meas}	0.140 (2.547) [a]
**Refinement statistics**	
Resolution limits	47.87-1.73
R_{work}/R_{free}	0.192/0.235
Average B-factor (Å^2)	37.3
Root mean square deviations	
Bond lengths (Å)	0.006
Bond angles (°)	0.89
Ramachandran statistics (%)	
Favored	97.5
Allowed	2.5
Outliers	0.0

[a] Numbers in parentheses refer to the highest resolution data shell

Supplementary Table 2. NOE R-factors for the CORCEMA-ST predictions based on the selected binding pose from the mixed-solvent MD simulations

ID	NOE R-factor (ND1)	NOE R-factor (N)
TROLL2	0.48	0.34
TROLL7	0.63	0.65
TROLL8	0.74	0.78
TROLL12	0.53	0.45

Supplementary Table 3. Comparison of the identified fragments in the screenings with the ND1-construct and the N-domain, respectively.

In addition, the mean and maximum STD-effect obtained in the NMR experiment with the ND1-construct is reported. ***n.b.*** no binding signal observed; ***n.d.*** affinity not determinable due to weak signal or poor signal quality; **-** no measurement carried out.

ID	structure	K_D [µM] (ND1)	K_D [µM] (N)	mean / maximum STD-effect (ND1)
TROLL1	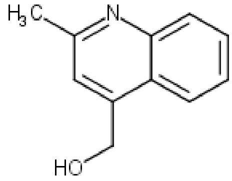	n.b.	37±3	18.0 / 22.5
TROLL2	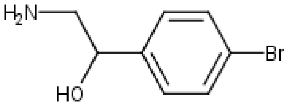	321±67	56±46	36.0 / 37.7
VIK40	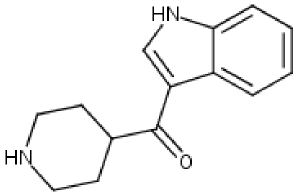	707±32	93±4	43.6 / 51.4
TROLL4	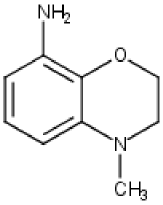	292±135	124±29	20.5 / 24.2
VIK20	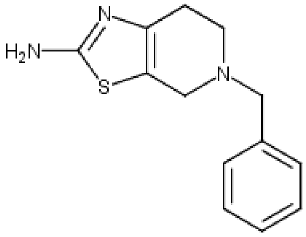	192±92	147±32	18.3 / 18.3
TROLL6	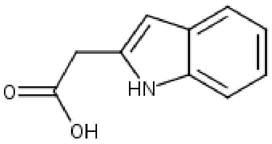	243±16	160±22	66.5 / 72.5
TROLL7	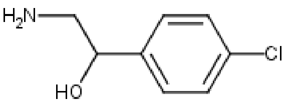	718±57	222±83	34.5 / 37.3
TROLL8	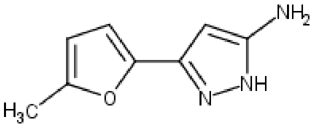	598±214	245±43	33.1 / 41.0

ID	structure	K_D [µM] (ND1)	K_D [µM] (N)	mean / maximum STD-effect (ND1)
TROLL9	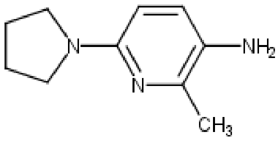	351±82	267±65	10.5 / 10.8
TROLL10	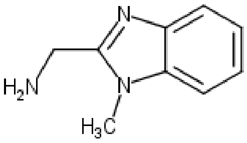	66±14	276±89	n.b.
TROLL11	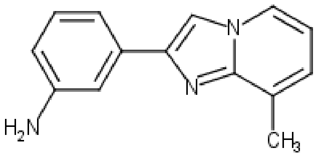	290±179	323±48	37.8 / 43.7
TROLL12	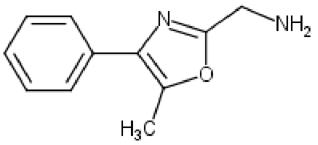	192±92	354±17	44.5 / 47.9
TROLL14	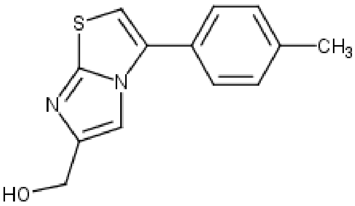	290±33	409±61	55.4 / 67.4
TROLL13	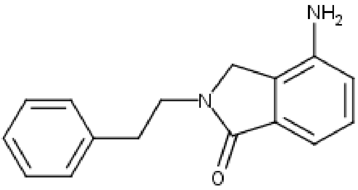	784±118	409±80	33.0 / 38.7
TROLL15	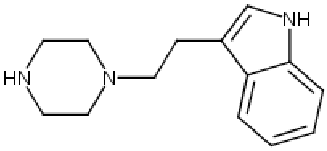	303±82	425±31	25.2 / 32.1
TROLL16	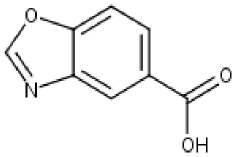	n.d.	440±53	n.b.
TROLL17	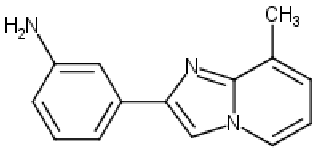	n.b.	444±56	11.3 / 12.5
TROLL18	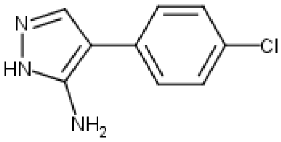	445±223	625±89	54.3 / 57.4

ID	structure	K_D [µM] (ND1)	K_D [µM] (N)	mean / maximum STD-effect (ND1)
TROLL19	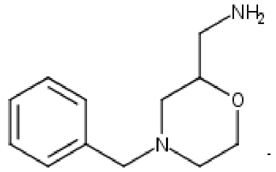	541±25	641±125	n.b.
TROLL20	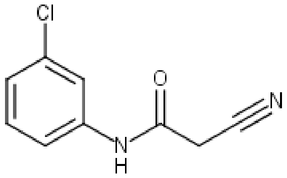	n.d.	714±525	-
TROLL21	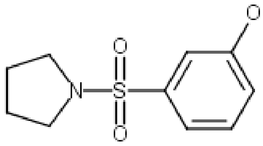	62±31	821±101	-
VIK50	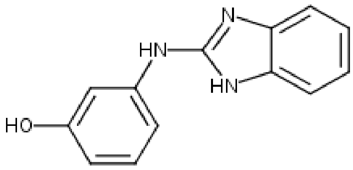	192±92	853±57	73.9
VIK80	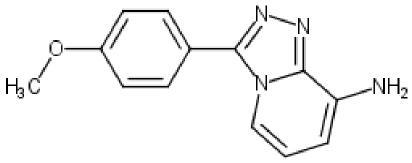	112±16	-	-
VIK1	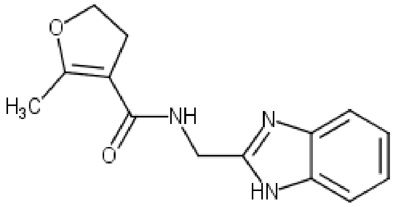	115±13	-	-
ODIN27	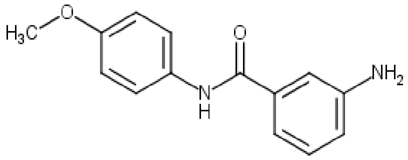	143±5	-	-
ODIN32	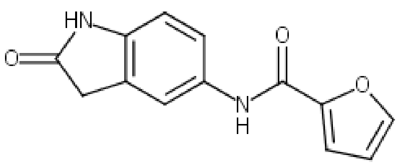	178±20	-	-
ODIN33	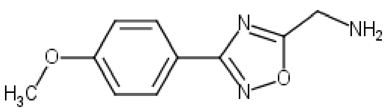	281±19	-	-

ID	structure	K_D [µM] (ND1)	K_D [µM] (N)	mean / maximum STD-effect (ND1)
ODIN26	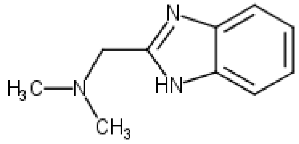	290±170	-	-
ODIN10	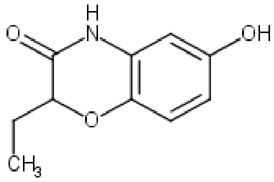	367±58	-	-
VIK110	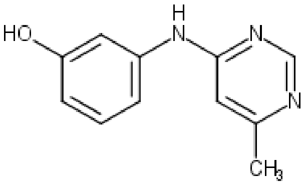	428±33	-	-
ODIN18	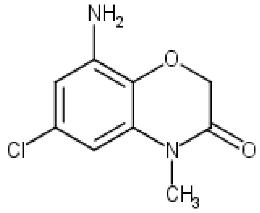	657±89	-	-
ODIN13	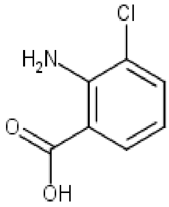	723±57	-	-
VIK90	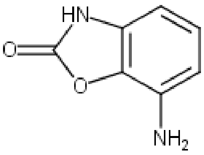	963±408	-	-

Sorted by K_D (N), and subsequently by K_D (ND1).

Supplementary Table 4. Small molecule screening data

Category	Parameter	Description
Assay	Type of assay	Optical biosensor (biolayer interferometry)
	Target	AAA+ p97
	Primary measurement	Assay validation by known positive binder (ADP)
	Key reagents	None
	Assay protocol	In-house
	Additional comments	No
Library	Library size	679
	Library composition	Subset of OTAVA chemicals solubility library
	Source	OTAVA chemicals
	Additional comments	No
Screen	Format	96 well plates
	Concentration(s) tested	Primary screen 500 µM, concentration dependencies 6 point 1:1 dilution series starting from 1 mM.
	Plate controls	Positive controls bevor and after each screening plate (p97 ND1)
	Reagent/ compound dispensing system	Mosquito HV, TTP Labtech
	Detection instrument and software	Sartorius Octet RED96e, raw data processing device software, follow up data processing with Origin Pro and R.
	Assay validation/QC	Z-factor (0.67)
	Correction factors	None
	Normalization	Yes, signals were normalized against ADP signal (p97 ND1), loading signal (p97 N), respectively.
	Additional comments	None

Category	Parameter	Description
Post-HTS analysis	Hit criteria	> 1.0 sigma
	Hit rate	29 (4.3%) for p97 ND1, 22 (3.2%) for p97 N
	Additional assay(s)	STD-NMR, ^{15}N-HSQC NMR, X-ray crystallography
	Confirmation of hit purity and structure	^{1}H-NMR
	Additional comments	None

Supplementary Methods

Definition of the quality score "Score$_{BLI}$"

For selecting fragments for further analysis, the $\mathrm{Score}_{\mathrm{BLI}}$ was defined as:

$$Score_{BLI} = \frac{r^2_{mean\ 1:1}}{r^2_{mean\ 2:1}}\, r^2_{kobs} - 100 \cdot |m_{Plateau}|$$

$\mathrm{r}^2_{\mathrm{mean\ 1:1}}$ is the mean of the r^2 coefficients of the regressions of all association phases measured for a given fragment. The data were fitted with a 1:1 binding model:

$$R_t = R_{eq}\left(1 - e^{-(kobs\ t)}\right)$$

with R_t the shift at the time t, k_{obs} the empirical rate constant and R_{eq} the shift at equilibrium. In analogy, the $\mathrm{r}^2_{\mathrm{mean\ 2:1}}$ was defined by fitting with a 2:1 binding model:

$$R_t = R_{eq\ 1}\left(1 - e^{-(kobs1\ t)}\right) + R_{eq\ 2}\left(1 - e^{-(kobs2\ t)}\right)$$

A ligand following a 1:1 model would result in a good $\mathrm{r}^2_{\mathrm{mean}}$ for both functions, whereas for a ligand which is better described with a 2:1 model the $\mathrm{r}^2_{\mathrm{mean}}$ for a 1:1 model would decrease, resulting in an overall decreased value.

In case of a 1:1 model the empirical rate constant $\mathrm{k}_{\mathrm{obs}}$ is proportional to the concentration of the ligand [L]:

$$k_{obs} = k_a[L] + k_d$$

with k_a the association rate constant and k_d the dissociation rate constant. Therefore, all observed empirical rate constants of a ligand were subjected to a linear regression model, which yielded the value $\mathrm{r}^2_{\mathrm{kobs}}$.

A Langmuir model was used to estimate the affinities of the fragments. The use of this model requires that a chemical equilibrium is reached. To analyze whether this condition was met, the responses of the last 10 seconds of each association phase were fitted with a linear model. At chemical equilibrium, the slope should be zero. The obtained slopes of each concentration were plotted against the logarithm of the molar concentration and fitted with a linear model. If the equilibrium was successfully reached for all concentrations, the resulting slope $\mathrm{m}_{\mathrm{plateau}}$ should also be close to zero, whereas unspecific binding, where no chemical equilibration can be observed, will lead to a slope > zero. The resulting value $\mathrm{m}_{\mathrm{plateau}}$ was used as a "penalty term". The calculated $\mathrm{Score}_{\mathrm{BLI}}$ should be close to or above 1.0 for a good 1:1 ligand, whereas ligands showing unspecific binding should yield values < 1.0. The score was evaluated with binding data for ADP, which gave a $\mathrm{Score}_{\mathrm{BLI}}$ of 1.02, and for a clearly non-specifically binding fragment (TROLL9), which gave a $\mathrm{Score}_{\mathrm{BLI}}$ of -0.15.

STD effect predictions using CORCEMA-ST

For the prediction of STD effects based on the identified binding modes in the mixed-solvent MD simulations, a representative pose of each fragment in complex with the protein (N domain) was selected as described in the Materials and Methods section. For each of these complex structures the ^{1}H chemical shifts of the protein were predicted using the online version of ShiftX V1.1 [3]. For the CORCEMA-ST [4] calculations the protein and ligand concentrations were used as described for the experimental STD-NMR measurements, and as affinities the results from the BLI measurements were used. For the k_{on} parameter we assumed a diffusion-limited rate of 10^9 $s^{-1}M^{-1}$. The irradiation range was set to 0 to 1.0 ppm, the τ-ligand was assumed with 0.5 ns and the τ-bound with 13 ns based on other protein rotational correlation times from spherical proteins in the same molecular weight range. In analogy to the STD-NMR experiments, the saturation times were set to 0.5, 0.75, 1.0, 1.5, 2.0, 3.0, and 5.0 s, respectively. For chemically equivalent protons the average saturation effect of those protons was calculated. The initial rate of STD build-up (STD_0) was obtained by fitting the data to the mono-exponential function:

$$STD_{tsat} = STD_{max} \cdot (1 - e^{-k_{sat} \cdot t_{sat}})$$

Here STD_{tsat} is the STD intensity at saturation time t_{sat}, STD_{max} is the equilibrium STD intensity, and k_{sat} is the rate constant of saturation transfer. The STD_0 effect was then calculated as follows:

$$STD_0 = \lim_{t \to 0} \frac{\Delta STD(t)}{\Delta t} = STD_{max} \cdot k_{sat}$$

Since the MD simulations were conducted only with the N-domain of p97 and the experiments were conducted with the full hexameric ND1 construct as well as the isolated N-domain, normalized STD_0 values were used (with normalization against the highest observed STD effect) for better comparison and further inspection [5].

For evaluation of the predicted STD_0 intensities the NOE R-factor was calculated for each model according to the following equation [6]:

$$NOE(R - factor) = \sqrt{\frac{\sum W_k (STD_{0,k\,exp.} - STD_{0,k\,pred.})^2}{\sum W_k (STD_{0,k\,exp.})^2}}$$

$STD_{0,k\ exp.}$ represents the experimental initial slope STD values and $STD_{0,k\ pred.}$ the predicted initial slope STD values. W_k is a weighting factor which was set to 1 because of normalized values being employed. NOE(R-factor) values of 0.0 represent a total agreement between model and experimental data, whereas 1.0 corresponds to a maximum discrepancy.

Chemical identity

TROLL2 (racemic mixture), purity > 95 %:

^{1}H NMR (400 MHz, DMSO): δ 7.49 (d, J = 8.4 Hz, 2H), 7.27 (d, J = 8.2 Hz, 2H), 5.31 (d, J = 3.5 Hz, 1H), 4.42 (m, 1H), 2.66-2.54 (m, 2H), 1.49 (s, 2H)

TROLL7 (racemic mixture, chloride salt), purity > 95 %:

^{1}H NMR (400 MHz, DMSO): δ 7.90 (s, 3H), 7.45 (d, J = 8.6 Hz, 2H), 7.41 (d, J = 8.7 Hz, 2H), 6.14 (d, J = 4.1 Hz, 1H), 4.79 (m, 1H), 3.06-2.80 (m, 2H)

TROLL8 (chloride salt), purity > 95 %:

^{1}H NMR (400 MHz, DMSO): δ 6.84 (d, J = 3.2 Hz, 1H), 6.26 (d, J = 3.2 Hz, 1H), 6.04 (s, 1H), 2.34 (s, 3H)

TROLL12, purity > 95 %::

^{1}H NMR (400 MHz, D_2O): δ 7.50 (d, J = 7.7 Hz, 2H), 7.40 (t, J = 7.8 Hz, 2H), 7.26 (t, J = 7.5 Hz, 1H), 3.75 (s, 2H), 2.27 (s, 3H)

Supplementary References

1. Chou, T.-F. *et al.* Specific Inhibition of p97/VCP ATPase and Kinetic Analysis Demonstrate Interaction between D1 and D2 ATPase domains. *J. Mol. Biol.* **426**, 2886–2899 (2014).

2. Isaacson, R. L. *et al.* Detailed structural insights into the p97-Npl4-Ufd1 interface. *J. Biol. Chem.* **282**, 21361–21369 (2007).

3. Neal S., Nip A. M., Zhang H. & Wishart D. S. Rapid and accurate calculation of protein 1H, 13C and 15N chemical shifts. *J. Biomol. NMR*, **26**(3), 215-240 (2003).

4. Jayalakshmi V. & Krishna N. R. Complete relaxation and conformational exchange matrix (CORCEMA) analysis of intermolecular saturation transfer effects in reversibly forming ligand-receptor complexes. *J. Magn. Reson*., **155**(1), 106-118 (2002).

5. Walpole, S., Monaco, S., Nepravishta, R. & Angulo, J. STD-NMR as a Technique for Ligand Screening and Structural Studies. *Methods in Enzymology*, **615**, 423-451 (2019).

6. Angulo, J., Díaz, I., Reina, J.J., Tabarani, G., Fieschi, F., Rojo, J. & Nieto, P.M. Saturation Transfer Difference (STD) NMR Spectroscopy Characterization of Dual Binding Mode of a Mannose Disaccharide to DC-SIGN. *Chem. Bio. Chem.*, **9**, 2225-2227 (2008).
