# Supplementary material for: Fragment screening using biolayer interferometry reveals ligands targeting the SHP-motif binding site of the AAA+ ATPase p97
Source: Commun Chem. 2022 Dec 7;5:169. doi: 10.1038/s42004-022-00782-5 (PMC9814400; doi:10.1038/s42004-022-00782-5)
Supplement: Supplementary file 2 — Reporting Summary [file 42004_2022_782_MOESM2_ESM.pdf]

nature portfolio

Corresponding author(s): Christoph Sotriffer, Hermann Schindelin

Last updated by author(s): Nov 10, 2022

Reporting Summary

Nature Portfolio wishes to improve the reproducibility of the work that we publish. This form provides structure for consistency and transparency in reporting. For further information on Nature Portfolio policies, see our Editorial Policies and the Editorial Policy Checklist.

Statistics

For all statistical analyses, confirm that the following items are present in the figure legend, table legend, main text, or Methods section.

n/a	Confirmed	
☐	☒	The exact sample size (*n*) for each experimental group/condition, given as a discrete number and unit of measurement
☐	☒	A statement on whether measurements were taken from distinct samples or whether the same sample was measured repeatedly
☐	☒	The statistical test(s) used AND whether they are one- or two-sided<br>*Only common tests should be described solely by name; describe more complex techniques in the Methods section.*
☒	☐	A description of all covariates tested
☐	☒	A description of any assumptions or corrections, such as tests of normality and adjustment for multiple comparisons
☐	☒	A full description of the statistical parameters including central tendency (e.g. means) or other basic estimates (e.g. regression coefficient) AND variation (e.g. standard deviation) or associated estimates of uncertainty (e.g. confidence intervals)
☒	☐	For null hypothesis testing, the test statistic (e.g. *F*, *t*, *r*) with confidence intervals, effect sizes, degrees of freedom and *P* value noted<br>*Give P values as exact values whenever suitable.*
☒	☐	For Bayesian analysis, information on the choice of priors and Markov chain Monte Carlo settings
☒	☐	For hierarchical and complex designs, identification of the appropriate level for tests and full reporting of outcomes
☒	☐	Estimates of effect sizes (e.g. Cohen's *d*, Pearson's *r*), indicating how they were calculated

Our web collection on statistics for biologists contains articles on many of the points above.

Software and code

Policy information about availability of computer code

Data collection	Biolayer interferometry data was collected using Sartorius Octet RED96e control software (version 9.0).<br>NMR data was collected using TopSpin (Bruker Biospin).<br>Mixed solvent MD-simulations were set up using GROMACS (version 2019.01).<br>X-ray data was collected using MXCuBE.
Data analysis	Biolayer interferometry data was processed using Sartorius Octet RED96e analysis software.<br>Screening data was further analyzed using in house python and R-scripts (code can be provided by the corresponding authors upon request).<br>Affinity data was analyzed using Origin Pro 2019b.<br>NMR data was analyzed using TopSpin (Bruker Biospin).<br>Mixed solvent MD-simulations was analyzed using Visual Molecular Dynamics (VMD) (Version 1.9.3).<br>Chemoinformatic analyses and structure-based analyses were carried out using MOE (Molecular Operating Environment, 2019.01, Chemical. Computing Group).<br>X-ray data was processed and analyzed using XDS, SHELX, AnoDe, CCP4 Suite and Phenix.

For manuscripts utilizing custom algorithms or software that are central to the research but not yet described in published literature, software must be made available to editors and reviewers. We strongly encourage code deposition in a community repository (e.g. GitHub). See the Nature Portfolio guidelines for submitting code & software for further information.

Data

Policy information about availability of data

All manuscripts must include a data availability statement. This statement should provide the following information, where applicable:

- Accession codes, unique identifiers, or web links for publicly available datasets
- A description of any restrictions on data availability
- For clinical datasets or third party data, please ensure that the statement adheres to our policy

The datasets generated during and/or analysed during the current study are available under https://doi.org/10.5281/zenodo.7300018.
All additional data are available from the corresponding authors on reasonable request.
All structural data is deposited and released by the PDB (7PUX).

Human research participants

Policy information about studies involving human research participants and Sex and Gender in Research.

Reporting on sex and gender	Does not apply to our study
Population characteristics	Does not apply to our study
Recruitment	Does not apply to our study
Ethics oversight	Does not apply to our study

Note that full information on the approval of the study protocol must also be provided in the manuscript.

Field-specific reporting

Please select the one below that is the best fit for your research. If you are not sure, read the appropriate sections before making your selection.

☒ Life sciences ☐ Behavioural & social sciences ☐ Ecological, evolutionary & environmental sciences

For a reference copy of the document with all sections, see nature.com/documents/nr-reporting-summary-flat.pdf

Life sciences study design

All studies must disclose on these points even when the disclosure is negative.

Sample size	Does not apply to our study
Data exclusions	Does not apply to our study
Replication	One point concentration measurements in biolayer interferometry assay were run in duplicate. Affinity data of fragments was calculated based on 6 point dilution series run in duplicate.
Randomization	Does not apply to our study
Blinding	Does not apply to our study

Reporting for specific materials, systems and methods

We require information from authors about some types of materials, experimental systems and methods used in many studies. Here, indicate whether each material, system or method listed is relevant to your study. If you are not sure if a list item applies to your research, read the appropriate section before selecting a response.

Materials & experimental systems

n/a	Involved in the study
☒	☐ Antibodies
☒	☐ Eukaryotic cell lines
☒	☐ Palaeontology and archaeology
☒	☐ Animals and other organisms
☒	☐ Clinical data
☒	☐ Dual use research of concern

Methods

n/a	Involved in the study
☒	☐ ChIP-seq
☒	☐ Flow cytometry
☒	☐ MRI-based neuroimaging
